# Peripheral arterial endothelial dysfunction predicts future cardiovascular events in diabetic patients with albuminuria: a prospective cohort study

**DOI:** 10.1186/s12933-020-01062-z

**Published:** 2020-06-13

**Authors:** Bo Kyung Koo, Woo-Young Chung, Min Kyong Moon

**Affiliations:** 1 Department of Internal Medicine, Seoul National University College of Medicine, Seoul, Republic of Korea; 2 Division of Endocrinology, Department of Internal Medicine, Seoul National University Boramae Medical Center, 20, Boramaero-5-gil, Dong-jak gu, Seoul, 07061 Korea; 3 Division of Cardiology, Department of Internal Medicine, Seoul National University Boramae Medical Center, Seoul, Republic of Korea

**Keywords:** Reactive hyperemia, Endothelial function, Type 2 diabetes, Ischemic heart disease, Nonfatal stroke, Heart failure, Chronic kidney disease

## Abstract

**Background:**

Reactive hyperemia-peripheral arterial tonometry (RH-PAT) is a noninvasive and simple test for evaluating the endothelial function. There has been sparse evidence on the usefulness of the RH-PAT index (RHI) in predicting future cardiovascular diseases among diabetic patients.

**Methods:**

Asymptomatic diabetic patients with albuminuria were selected; their medical history and laboratory findings were evaluated every 3 to 4 months, respectively. The primary outcome was a composite of three-point major adverse cardiovascular events (3-point MACE): death from cardiovascular causes, acute coronary events, or nonfatal stroke. On the contrary, secondary outcomes included a composite of 3-point MACE, hospitalization for heart failure, or chronic kidney disease (CKD) progression. RHI was measured using the Endo-PAT2000 at the baseline. RHI < 1.67 was considered to indicate peripheral endothelial dysfunction (PED).

**Results:**

In total, 149 subjects were included (mean age, 61.8 ± 9.2 years; duration of diabetes was 12 years). During the follow-up period (median, 49.7 months), of the 149 subjects, primary outcomes were detected in 12 (1 [2.3%] and 11 [10.5%] of those without and with PED, respectively). The presence of PED in baseline measurements significantly increased both primary and secondary outcomes, following adjustment for age, sex, hypertension, glycated hemoglobin, low-density lipoprotein cholesterol, triglyceride, systolic blood pressure, baseline estimated glomerular filtration rate, overt proteinuria, duration of diabetes, premedical history of ischemic events, anti-platelet agents, and smoking history (hazard ratio [HR]: 10.95; 95% confidence interval CI 1.00–119.91 for the primary outcome; HR, 4.12; 95% CI 1.37–12.41 for secondary outcome). In addition, PED could predict secondary outcomes independent of the risk score according to the American College of Cardiology/American Heart Association (HR: 3.24; 95% CI 1.14–9.17).

**Conclusions:**

PED can independently predict future cardiovascular events among diabetic patients with albuminuria.

## Background

The leading cause of death in diabetic patients is atherosclerotic cardiovascular disease (ASCVD) [1]. However, intensive interventions targeting multifactorial ASCVD risk factors in the diabetic population have decreased vascular complications and mortality rates, [2] which weakened the prediction power of risk prediction based on traditional ASCVD risk factors [3, 4]. Currently, no risk scoring system has been developed based on diabetic patients on optimal medical treatment.

Reactive hyperemia-peripheral arterial tonometry (RH-PAT) is a simple, noninvasive, automatic test for evaluating the endothelial function [5, 6]. RH-PAT index (RHI) has been reported to be appropriately correlated with flow-mediated dilatation [5] and endothelin-1,[7] which are reliable markers of endothelial function. As RHI directly reflects endothelial function, it might estimate the residual ASCVD risk in diabetic subjects under appropriate medical treatment. Furthermore, endothelial dysfunction precedes atherosclerosis, [8] and RHI can predict the presence of ASCVD itself [9, 10]. Prospective studies confirmed that low levels of RHI could predict future cardiovascular events independently in the general population [11, 12]. However, there has been sparse evidence on the predictability of RHI in addition to conventional risk factors for future ASCVD among diabetic patients. Only a small-sized cross-sectional study [13] and a prospective study (less than 2 years) [14] reported no differences in RHI between the diabetic patients with and without coronary artery disease (CAD).

Diabetes itself is an important risk factor for ASCVD [14]. As albuminuria is a well-known risk factor for endothelial dysfunction [15] and ASCVD, [16] diabetic subjects with albuminuria consequently have a high risk of ASCVD. In this study, asymptomatic diabetic patients with albuminuria had their RHI levels evaluated, and future cardiovascular events were prospectively assessed. In addition, it was investigated whether RHI can provide any information alongside established ASCVD risk factors including Pooled Cohort Equations (PCE) for ASCVD according to the American College of Cardiology/American Heart Association [17].

## Methods

### Study subjects

This was a prospective cohort study of type 2 diabetic patients with albuminuria. Eligible patients with type 2 diabetes were: (i) ≥ 18 years of age, (ii) urinary albumin-to-creatinine ratio (ACR) ≥ 30 mg of albumin per gram of creatinine; and (iii) estimated glomerular filtration rate (eGFR) ≥ 30/min/1.73 m^2^ as recommended by the Modification of Diet in Renal Disease (MDRD) criteria [18]. Patients were excluded if they had (i) a recent history of myocardial infarction, cerebral infarction, or hospitalization due to heart failure less than 3 months before the enrollment, (ii) typical anginal pain aggravated by exercise, (iii) any chest discomfort accompanied with dyspnea, or (iv) grade 3 hypertension (systolic blood pressure (SBP) ≥ 180 mmHg or diastolic blood pressure (DBP) ≥ 110 mmHg) [19].

The cohort subjects were prospectively enrolled, and their RHI levels were measured within 3 months of enrollment. Their medical history and laboratory findings were followed every 3 or 4 months; cardiovascular and renal outcome events and deaths were prospectively monitored. The protocol was approved by the Institutional Review Board of the Seoul National University Boramae Medical Center, and written informed consent was obtained from all participants.

### Measurement of reactive hyperemic index

RHI was measured using the Endo-PAT2000 (Itamar Medical Ltd., Caesarea, Israel) similar to other previous studies [20, 21]. Briefly, after resting for at least 15 min, the pressure cuff on the forearm was inflated and maintained at 50 mmHg above the SBP to occlude the brachial artery. The cuff was deflated 5 min later, and RHI was automatically calculated by an internal algorithm based on pulse wave amplitude at the baseline and 1 min after deflation. RHI < 1.67 was considered to indicate peripheral arterial endothelial dysfunction (PED) [20, 21].

### Study outcomes

The primary outcome was a composite of three major adverse cardiovascular events (3-point MACE) which were defined as follows: death from cardiovascular causes, acute coronary events, or nonfatal stroke. Secondary outcomes included a composite of 3-point MACE, hospitalization for heart failure, or chronic kidney disease (CKD) progression.

An acute coronary event was defined as hospitalization for unstable angina or nonfatal myocardial infarction. The onset or worsening of CKD was defined as follows: (i) a decrease from baseline in eGFR by 30% or more to an eGFR < 60 mL/min per 1.73 m^2^, (ii) an eGFR < 30 mL/min per 1.73 m^2^, (iii) the initiation of renal-replacement therapy, or (iv) death from renal disease.

### Evaluation for ASCVD risk factors

Plasma glucose and lipid concentrations were measured enzymatically using a Hitachi Automatic Analyzer B2400 (Hitachi, Tokyo, Japan), and glycated hemoglobin (HbA1c) levels were measured using a 200FR chemistry analyzer (Toshiba, Tokyo, Japan). Serum creatinine levels were measured every 3–6 months using an assay based on isotope dilution mass spectrometry. The patients’ eGFR was calculated using the MDRD Study equation [18].

Sex-specific PCE for non-Hispanic whites that estimates the 10-year risk of ASCVD according to the American College of Cardiology/American Heart Association [17] was also used to adjust conventional ASCVD risk, as no specific equation exists for Koreans.

### Statistical analysis

All data were analyzed using IBM SPSS Statistic 20.0 for Windows (IBM Inc., Chicago, IL, USA). Demographic and clinical data between those with and without PED were compared with the Mann–Whitney test, an independent *t* test, and a Chi square test. The Cox proportional hazards model was used to investigate the predicting factors for primary or secondary outcomes with adjustments for sex, age, hypertension, HbA1c, low-density lipoprotein (LDL) cholesterol, triglyceride, overt proteinuria (ACR ≥ 300 mg of albumin per gram of creatinine), baseline e-GFR, premedical history of ischemic events, duration of diabetes, anti-platelet agents, and smoking history. The independent determining factors for PED (RHI < 1.67) were investigated using backward multivariable logistic regression. The level of significance was set at *P* < 0.05.

In the previous study, 16% of diabetic subjects with albuminuria experienced MACE during 3.1 years of follow-up [22]; and previous studies based on general population reported approximately 20% difference in ASCVD event according to the absence or presence of PED [11, 12]. With 0.05, 0.20 and 0.80 of α error, β error, and power, respectively, 148 subjects were needed for the study.

## Results

### Baseline characteristics

In total, 149 subjects were included; the median follow-up period was 49.7 months (range: 3–69 months). At the baseline, mean age was 61.8 ± 9.2 years, and median duration of diabetes was 12 years (interquartile range [IQR], 7–17 years) (Table 1). Among them, 105 subjects (70.5%) had PED. These subjects were significantly older than those without PED (62.8 ± 8.7 years vs. 59.4 ± 9.9; *P* = 0.043). Despite no difference in the prevalence of hypertension or the proportion of subjects on angiotensin receptor blocker (ARB) or angiotensin-converting enzyme inhibitor (ACEI) between the two groups, subjects with PED experienced low SBP and DBP compared to those without PED (Table 1). The proportion of subjects taking statin at a moderate dose or more [23] was 67.6% and 75.0% in those with and without PED, respectively (no difference between groups; *P* = 0.371). Overall, only 9 subjects took sodium-glucose cotransporter 2 (SGLT2) inhibitors, whereas no subject took glucagon-like peptide 1 receptor analogue (GLP1-RA) at the baseline (Table 1). No difference was observed in body mass index (BMI), HbA1c, lipid profile, eGFR, or ACR. The presence or absence of PED was not an indicator of previous vascular events; however, subjects with PED more frequently used anti-platelet agents than subjects with RHI ≥ 1.67 (52.4% vs. 31.8%; *P* = 0.022).

**Table 1:** Baseline clinical characteristics according to peripheral endothelial dysfunction (PED)

	Total (n = 149)	No PED (n = 44)	PED (n = 105)	*P*^1^
Age, year	61.8 ± 9.2	59.4 ± 9.9	62.8 ± 8.7	0.043
Men, n (%)	79 (53.0)	23 (52.3)	56 (53.3)	0.906
Duration of DM, years	12.0 (7.0–17.0)	11.0 (7.0–16.0)	13.0 (7.5–18.5)	0.309
BMI, kg/m^2^	26.1 (24.1–28.0)	26.9 (24.6–28.4)	26.0 (23.9–27.5)	0.087
SBP, mmHg	130.4 ± 15.4	134.4 ± 15.2	128.8 ± 15.2	0.042
DBP, mmHg	77.2 ± 10.6	80.2 ± 12.7	75.9 ± 9.3	0.045
Hypertension, n (%)	134 (89.9)	39 (88.6)	95 (90.5)	0.733
HbA1c, %	7.2 (6.7–7.8)	7.1 (6.8–7.5)	7.2 (6.7–7.9)	0.636
LDL cholesterol, mg/dL	81.0 (68.5–98.0)	81.0 (69.0–97.8)	80.0 (67.0–94.0)	0.499
HDL cholesterol, mg/dL	44.0 (37.0–52.0)	43.5 (36.3–55.0)	44.0 (37.0–50.5)	0.528
Triglyceride, mg/dL	125.0 (88.0–176.5)	108.0 (78.5–156.3)	132.0 (95.5–182.5)	0.057
hsCRP, mg/L	0.6 (0.3–1.4)	0.7 (0.4–1.3)	0.6 (0.3–1.6)	0.850
eGFR, mL/min/1.73 m^2^	79.1 (62.2–92.9)	83.8 (69.1–92.7)	77.8 (61.4–93.0)	0.295
eGFR ≥ 90, n (%)	46 (30.9)	14 (31.8)	32 (30.5)	0.583
eGFR 60–89, n (%)	77 (51.7)	24 (54.5)	53 (50.5)	
eGFR 30–59, n (%)	26 (17.4)	6 (13.6)	20 (19.0)	
ACR, mg/g	95.2 (49.9–235.3)	92.4 (50.7–210.5)	97.5 (45.0–251.5)	0.867
Overt proteinuria, n (%)	32 (21.5)	9 (20.5)	23 (21.9)	0.844
Diabetic retinopathy				
No	65 (47.4)	21 (51.2)	44 (45.8)	0.645
NDPR	44 (32.1)	12 (29.3)	32 (33.3)	
PDR	28 (20.4)	8 (19.5)	20 (20.8)	
Current smoker, n (%)	47 (31.5)	10 (22.7)	37 (35.2)	0.134
ARB or ACEI, n (%)	114 (80.3)	34 (79.1)	80 (80.8)	0.811
CCB, n (%)	65 (45.8)	22 (51.2)	43 (43.4)	0.396
BB, n (%)	15 (10.6)	7 (16.3)	8 (8.1)	0.144
Statin, n (%)	128 (85.9)	37 (81.8)	93 (87.6)	0.545
No statin	19 (12.8)	7 (15.9)	12 (11.4)	0.746
Low intensity	26 (17.4)	4 (9.1)	22 (21.0)	
Moderate intensity	102 (68.5)	32 (72.7)	70 (66.7)	
High intensity	2 (1.3)	1 (2.3)	1 (1.0)	
Anti-platelet, n (%)	69 (46.3)	14 (31.8)	55 (52.4)	0.022
Anti-diabetic drugs				
Metformin	140 (94.0)	41 (93.2)	99 (94.3)	0.796
SGLT2 inhibitors	9 (6.0)	4 (9.1)	5 (4.8)	0.312
SU or insulin	120 (80.5)	32 (72.7)	88 (83.3)	0.119
Previous IHD, n (%)	4 (2.7)	0	4 (3.8)	0.189
Previous stoke, n (%)	4 (2.7)	2 (4.5)	2 (1.9)	0.369

PED was defined as RHI < 1.67. All values are expressed in mean ± standard deviation or median (interquartile range) for continuous variables and proportions (%) for categorical variables

DM, diabetes mellitus; BMI, body mass index; SBP, systolic blood pressure; DBP, diastolic blood pressure; LDL, low-density lipoprotein; HDL, high-density lipoprotein; CRP, c-reactive protein; eGFR, estimated Glomerular filtration rate; ACR, albumin-to-creatinine ratio; NPDR, non-proliferative diabetic retinopathy; PDR, proliferative diabetic retinopathy; ARB, angiotensin receptor blocker; ACEI, angiotensin converting enzyme inhibitor; CCB, calcium channel blocker; BB, beta-blocker; SGLT2, sodium-glucose cotransporter 2; GLP1-RA, glucagon-like peptide 1 receptor analogue; SU, sulfonylurea; IHD, ischemic heart disease

^1^Comparison between those with and without PED using Mann-Whitney test, independent t test and chi-square test

### Determinants of the baseline RHI

To investigate the independent determinant of PED at the baseline, backward multivariable logistic regression was performed incorporating age, sex, BMI, HbA1c, SBP, DBP, hypertriglyceridemia, hypertension, smoking history, anti-platelet agents, and prescriptions of cilostazol or statin. In the final model, only age, SBP, hypertriglyceridemia, and smoking history showed a correlation (Additional file 1: Table S1). Age and current smoking were significantly associated with PED (odds ratio [OR], 1.06; 95% CI 1.02–1.11; *P* = 0.008; and OR, 2.97; 95% CI 1.15–7.63; *P* = 0.024, respectively). On the contrary, SBP showed a negative association with the risk of PED (OR 0.96; 95% CI 0.94–0.99; *P* = 0.006).

### Future cardiorenal events according to RHI

During the follow-up period (median 49.7 months), primary outcomes were detected in 12 of 149 subjects (1 [2.3%] and 11 [10.5%] in those without and with PED, respectively; *P* = 0.093; Table 2). No mortality was associated with cardiovascular causes. Secondary outcomes were detected in 37 of 149 subjects (4 [9.1%] vs. 33 [31.4%] in those without and with PED, respectively; *P* = 0.004).

**Table 2:** Cox Proportional Hazards Analysis for Cardiovascular Events according to Peripheral Endothelial Dysfunction (PED)

	Events, n (%)			Unadjusted		Model 1		Model 2	
	No PED (n = 44)	PED (n = 105)	P*	HR (95% CI)	*P*	HR (95% CI)	*P*	HR (95% CI)	*P*
Primary outcome	1 (2.3)	11 (10.5)	0.093	4.62 (0.60, 35.79)	0.143	11.49 (1.16, 114.24)	0.037	10.95 (1.00, 119.91)	0.050
Secondary outcome	4 (9.1)	33 (31.4)	0.004	3.45 (1.22, 9.75)	0.019	3.85 (1.31, 11.29)	0.014	4.12 (1.37, 12.41)	0.012
Acute coronary event	1 (2.3)	9 (8.6)	0.161	3.79 (0.48, 29.88)	0.207	9.24 (0.90, 95.36)	0.062	8.05 (0.64, 101.10)	0.106
Stroke	0	2 (1.9)	0.357	–	0.594	–	0.983	–	0.965
Heart failure	0	3 (2.9)	0.257	–	0.498	–	0.953	–	0.858
CKD^1^	4 (9.1)	23 (21.9)	0.064	2.16 (0.74, 6.26)	0.157	2.17 (0.72, 6.61)	0.171	3.26 (1.01, 10.50)	0.048

PED was defined as RHI < 1.67. Primary outcome was composed of 3-point major adverse cardiovascular events (MACE); secondary outcome was a composite of 3-point MACE, hospitalization for heart failure, or chronic kidney disease (CKD)

Model 1: adjusted for age, sex, hypertension, HbA1c, LDL cholesterol, triglyceride, proteinuria, duration of diabetes, and premedical history of ischemic events

Model 2: adjusted for systolic blood pressure, baseline e-GFR, anti-platelet agents, and smoking history in addition to Model 1

^1^CKD progression defined as decrease from baseline in eGFR by 30% or more to an eGFR of less than 60 mL/min per 1.73 m^2^, or an eGFR of less than 30 mL/min per 1.73 m^2^ during the follow-up period

^*^Comparison the number of events between those with and without PED

**Fig. 1:**
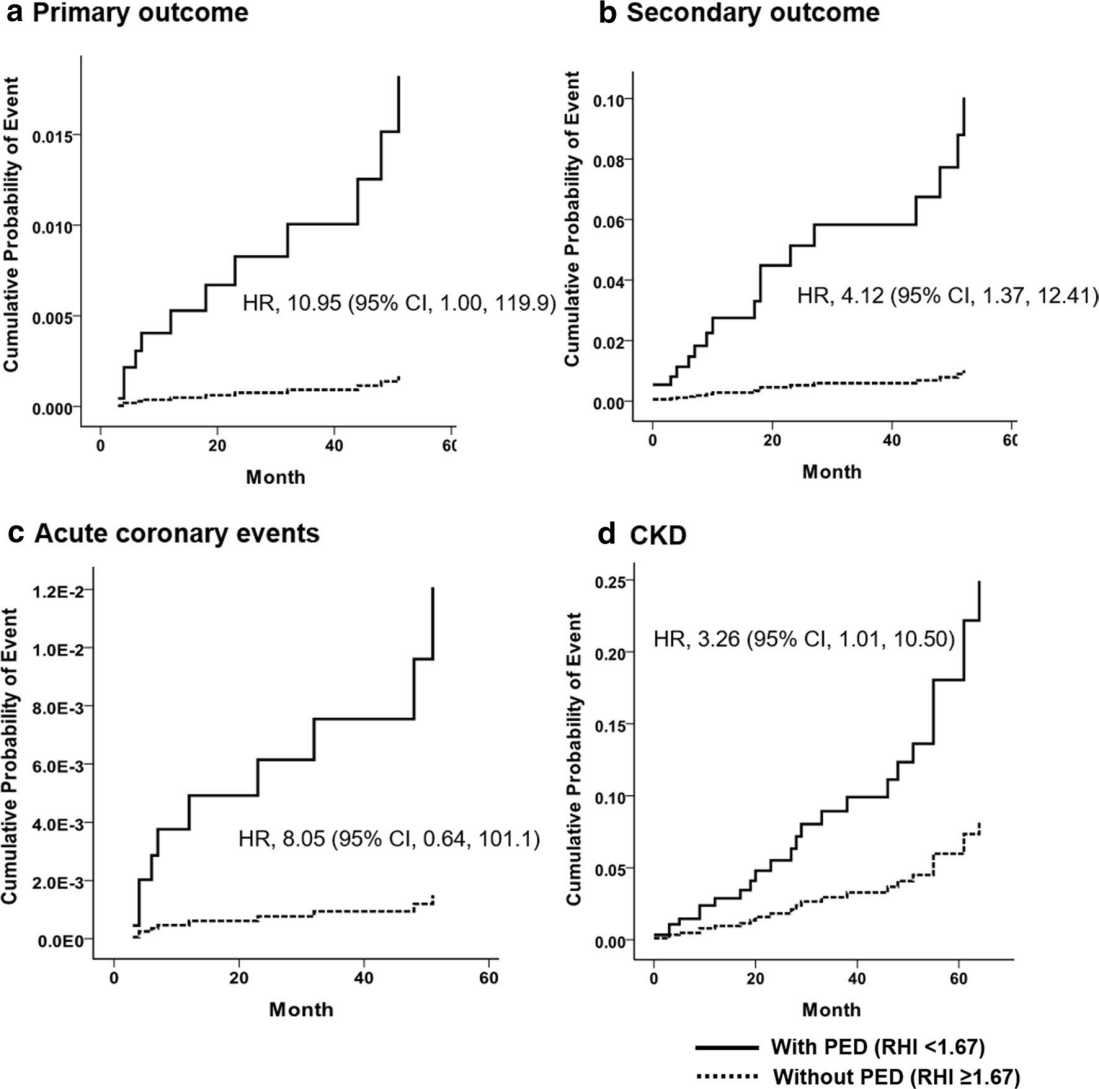
Cumulative probability of primary and secondary outcomes according to peripheral arterial endothelial dysfunction (PED). Cox proportional hazards model was used to investigate the effect of PED (RHI < 1.67) on the primary and secondary outcome event rates during the follow-up period with adjustment for sex, age, hypertension, glycated hemoglobin (HbA1c), low density lipoprotein (LDL) cholesterol, triglyceride, overt proteinuria, baseline estimated glomerular filtration rate (e-GFR), premedical history of ischemic events, duration of diabetes, anti-platelet agents, and smoking history. Solid and dashed lines represent cumulative probability of **a** primary outcome composed of 3-point major adverse cardiovascular events (MACE), **b** secondary outcome composed of 3-point MACE, hospitalization for heart failure, or chronic kidney disease (CKD) progression, **c** acute coronary events, and **d** CKD progression in those with and without PED, respectively. RHI, reactive hyperemia index; MACE, major adverse cardiovascular event; CKD, chronic kidney disease; e-GFR, estimated glomerular filtration rate; CAC, coronary artery calcification; ASCVD, atherosclerotic cardiovascular disease; CAD, coronary artery disease; SBP, systolic blood pressure; DBP, diastolic blood pressure; MDRD, Modification of Diet in Renal Disease; PED, peripheral endothelial dysfunction; PCE, pooled Cohort Equations

In unadjusted Cox-regression analysis, the presence of PED at the baseline did not increase the risk of the primary outcomes significantly (hazard ratio [HR], 4.62; 95% CI 0.60–35.79; *P* = 0.143). Subjects with PED experienced secondary outcomes more frequently than those without PED (HR, 3.45; 95% CI 1.22–9.75; *P* = 0.019; Table 2). After adjusting for age, sex, hypertension, HbA1c, LDL cholesterol, triglyceride, proteinuria, duration of diabetes, and premedical history of ischemic events, presence of PED increased both primary and secondary outcomes (Model 1 in Table 2). This trend persisted following additional adjustments for SBP, baseline e-GFR, anti-platelet agents, and smoking history for both primary (HR, 10.95; 95% CI 1.00–119.91; *P* = 0.050; Fig. 1a) and secondary outcomes (HR, 4.12; 95% CI 1.37–12.41; *P* = 0.012; Fig. 1b; Model 2 in Table 2).

Subsequent Cox-regression analysis incorporating PCE and PED confirmed PED as an independent risk factor for secondary outcomes (HR, 3.24; 95% CI 1.14–9.17; *P* = 0.027; Additional file 1: Table S2).

CKD progression was observed in 27 subjects during the follow-up period, which was associated with PED at the baseline in the fully adjusted model (HR, 3.26; 95% CI 1.01–10.50; *P* = 0.048; Model 2 in Table 2; Fig. 1). Among subjects experiencing secondary outcomes, those with CKD progression had significantly higher SBP (134.9 ± 15.6 mmHg vs. 118.9 ± 12.6 mmHg; *P* = 0.006) and lower eGFR (median eGFR, 61.2 vs. 82.1 mL/min/1.73 m^2^; *P* = 0.023) at the baseline than those without CKD progression. Notably, no difference in RHI at baseline was observed between them (Table 3).

**Table 3:** Baseline clinical characteristics according to CKD progression during the follow-up period

	Without any events	With any events			*P*^1^	*P*^2^
		Entire	No CKD progression	CKD progression		
	n = 112	n = 37	n = 10	n = 27		
Age, year	61.0 ± 9.3	64.1 ± 8.5	66.7 ± 8.5	63.6 ± 8.5	0.044	0.323
Men, n (%)	58 (51.8)	21 (56.8)	7 (70.0)	14 (51.9)	0.599	0.322
Duration of DM, years	11.0 (7.0–16.8)	14.0 (8.0–17.0)	10.0 (6.0–17.5)	15.0 (11.0–19.0)	0.242	0.319
BMI, kg/m^2^	26.4 (24.1–28.0)	25.8 (24.2–27.7)	25.2 (23.9–27.6)	25.8 (24.6–28.7)	0.654	0.468
SBP, mmHg	130.3 ± 15.0	130.8 ± 16.6	118.9 ± 12.6	134.9 ± 15.6	0.863	0.006
DBP, mmHg	78.2 ± 10.8	73.8 ± 9.2	72.6 ± 10.7	73.8 ± 8.6	0.017	0.722
Hypertension, n (%)	100 (89.3)	34 (91.9)	8 (80.0)	26 (96.3)	0.648	0.107
HbA1c, %	7.2 (6.8–7.8)	7.3 (6.7–8.0)	7.0 (6.6–8.0)	7.3 (6.6–8.0)	0.584	0.625
LDL cholesterol, mg/dL	81.0 (69.3–94.8)	77.0 (66.0–97.0)	70.5 (64.0–99.3)	78.0 (67.0–99.0)	0.597	0.408
HDL cholesterol, mg/dL	44.0 (37.0–51.8)	43.0 (38.0–53.0)	49.5 (39.5–55.0)	41.0 (33.0–53.0)	0.661	0.257
Triglyceride, mg/dL	123.5 (88.0–171.0)	127.0 (91.0–188.0)	111.0 (74.8–180.0)	127.0 (103.0–216.0)	0.333	0.271
hsCRP, mg/L	0.5 (0.3–1.3)	0.9 (0.4–2.4)	1.9 (0.6–3.3)	0.8 (0.3–1.9)	0.113	0.122
eGFR, mL/min/1.73 m^2^	84.7 (70.8–94.3)	61.5 (51.9–72.9)	82.1 (51.0–98.8)	61.0 (49.8–67.0)	< 0.001	0.023
ACR, mg/g	77.5 (44.9–146.4)	265.7 (79.1–636.6)	234.6 (52.7–778.6)	285.0 (174.7–835.4)	< 0.001	0.353
Overt proteinuria, n (%)	13 (11.6)	19 (51.4)	4 (40.0)	15 (55.6)	< 0.001	0.401
RHI	1.50 (1.31–1.85)	1.39 (1.26–1.57)	1.43 (1.23–1.48)	1.37 (1.26–1.59)	0.050	0.625
Diabetic retinopathy						
No	55 (53.4)	10 (29.4)	2 (22.0)	8 (32.0)	0.038	0.706
NDPR	29 (28.2)	15 (44.1)	6 (66.7)	9 (36.0)		
PDR	19 (18.4)	9 (26.5)	1 (11.1)	8 (32.0)		
Current smoker, n (%)	36 (32.1)	11 (29.7)	4 (40.0)	7 (25.9)	0.784	0.406
ARB, n (%)	86 (81.1)	28 (77.8)	6 (66.7)	22 (81.5)	0.662	0.355
CCB, n (%)	46 (43.4)	19 (52.8)	4 (44.4)	15 (55.6)	0.329	0.563
BB, n (%)	9 (8.5)	6 (16.7)	0	6 (22.2)	0.168	0.121
Statin, n (%)	98 (87.5)	30 (81.1)	8 (80.0)	22 (81.5)	0.331	0.919
Anti-platelet, n (%)	49 (43.8)	20 (54.1)	6 (60.0)	14 (51.9)	0.276	0.659
Previous IHD, n (%)	0	4 (10.8)	1 (10.0)	3 (11.1)	< 0.001	0.923
Previous stoke, n (%)	3 (2.7)	1 (2.8)	0	1 (3.7)	0.975	0.558

All values are expressed in mean ± standard deviation or median (interquartile range) for continuous variables and proportions (%) for categorical variables

CKD progression defined as decrease from baseline in eGFR by 30% or more to an eGFR of less than 60 mL/min per 1.73 m^2^, or an eGFR of less than 30 mL/min per 1.73 m^2^ during the follow-up period

CKD, chronic kidney disease; DM, diabetes mellitus; BMI, body mass index; SBP, systolic blood pressure; DBP, diastolic blood pressure; LDL, low-density lipoprotein; HDL, high-density lipoprotein; CRP, c-reactive protein; eGFR, estimated Glomerular filtration rate; ACR, albumin-to-creatinine ratio; NPDR, non-proliferative diabetic retinopathy; PDR, proliferative diabetic retinopathy; ARB, angiotensin receptor blocker; CCB, calcium chanel blocker; BB, beta-blocker; IHD, ischemic heart disease

^1^Compared between those without and without any events using Mann-Whitney test, independent t test and chi-square test

^2^Compared between those with and without CKD progression among subjects with any events using Mann-Whitney test, independent t test and chi-square test

## Discussion

In this study, 149 diabetic patients with albuminuria (median duration of diabetes was 12 years) were followed for 49.7 months, and the primary outcomes, defined as 3-point MACE, were detected in 8.1% of study subjects. PED (RHI < 1.67) was an independent risk factor for developing primary outcomes following adjustments for age, sex, hypertension, HbA1c, LDL cholesterol, triglyceride, SBP, baseline e-GFR, overt proteinuria, duration of diabetes, premedical history of ischemic events, anti-platelet agents, and smoking history. Subjects with PED at baseline had 10.95 times higher incidence of primary outcomes during the follow-up period in the fully adjusted model.

Similarly, PED was an independent risk factor for CKD progression in this study; subjects with PED showed 3.26 times higher risk of CKD progression. CKD shares common risk factors and pathophysiological mechanisms with ASCVD [24–26]. The combined risk of ASCVD and CKD was increased 4.12 times by PED at the baseline.

Traditionally, diabetes is regarded as a CAD equivalent [14]. However, intensive interventions targeting multifactorial ASCVD risk factors in the diabetic population have decreased vascular complications and mortality rates [2]. This resulted in a substantial reduction in death from cardiovascular causes, [27, 28] and weakened the prediction power of risk prediction based on traditional ASCVD risk factors [3, 4]. The estimation of the 10-year risk of ASCVD according to the American College of Cardiology/American Heart Association by PCE [17] was developed based on subjects enrolled in the 1970s-1990s; before the 1990s, statin therapy was not available. Accordingly, PCE overestimates ASCVD risk in modern cohorts [29]. In this study, 69.8% of the study subjects took statin with moderate intensity or more [23] at the baseline, and their baseline LDL cholesterol was 81.0 mg/dL. Currently, there is no risk scoring system developed based on diabetic patients under multifactorial treatment.

As the RHI directly reflects endothelial function regardless of the presence or absence of traditional ASCVD risk factors [11, 12], it could estimate the residual ASCVD risk independent of well-known ASCVD risk factors in diabetic subjects. In this study, PED could predict primary outcome only after adjusting ASCVD risk factors, which might be a supporting evidence for the possible estimation of the residual ASCVD risk by RHI. However, Venuraju S et al. recently reported that RHI could not predict MACE in diabetic patients [30]. Similarly, no difference was reported in coronary artery calcification (CAC) according to PED [30]. However, only a total of 18 MACE was observed in < 2 years of follow-up, and too small number of events for a short period might weaken the statistical significance. In addition, a wide distribution of baseline CAC observed in the study subjects reflects heterogeneity in ASCVD risk at the baseline among subjects. This might result not only in no association between CAC and RHI but also in no difference in MACE rate during the study period according to the PED status. However, considering that CAC reflects subclinical atherosclerosis and is useful for predicting ASCVD in asymptomatic diabetic patients along with the established cardiovascular risk factors, [31, 32], the determination of ASCVD risk by PED in addition to CAC should be further investigated [33–35].

Low RHI was reported not only in diabetes [13, 36–38] but also in metabolic syndrome [39] and nonalcoholic fatty liver disease [40]. Diabetic patients demonstrated lower RHI levels than the general population [13, 36, 37]. In this study, low RHI was detected in approximately 70% of the study subjects (105 out of 149 subjects), which was relatively higher than previous studies based on the general population where this was observed in 23–27% of the study subjects [20, 21]. In previous studies including diabetic patients, the mean RHI was 1.69; [13] a finding similar to ours. This study included subjects with albuminuria. Albuminuria is a well-known risk factor of endothelial dysfunction [15] and ASCVD, [16] which might result in a prevalent low RHI in this study.

At the baseline, no difference was observed in HbA1c, according to RHI in this study. In diabetic subjects, low RHI level was reported to be associated with poor glycemic control status [13, 30, 38]; however, there has been some controversies regarding this [41]. In addition, the median HbA1c in this study was 7.2 (IQR, 6.7–7.8), a relatively fair level, which might result in a negative finding in the association between glycemic level and RHI. On the contrary, lower SBP, age, and smoking were independent risk factors for low RHI in this study. However, no difference was observed in the prevalence of hypertension or the use of antihypertensive medication according to RHI. There is supporting information for a positive correlation, [42, 43] and a negative correlation [39, 44] between blood pressure and RHI. Heterogeneity in clinical characteristics of study subjects among studies might cause a difference in the relationship between RHI and blood pressure. In addition, inconsistent correlations between RHI and well-known ASCVD risk factors have been reported not only in blood pressure [30, 39, 42, 44–46] but also in age [39, 47, 48]. Age is one of the most important risk factors for ASCVD; however, RHI has been reported to be positively correlated [39, 47] and negatively correlated [48] with age. The association between RHI and blood pressure and/or age should be further investigated in large sized independent studies.

Anti-diabetic medications such as SGLT2 inhibitors or GLP1-RA may influence the endothelial function, [49] which was not observed in this study. At the baseline, only a small number of patients enrolled in this study were taking SGLT2 inhibitors (5 of 105 and 4 of 44 in those with and without PED, respectively), whereas no subject was on GLP1-RA. This study enrolled subjects from March 2013 to January 2017; in addition, the prescription of SGLT2 inhibitors and dulaglutide (the only long-acting GLP1-RA available in Korea) commenced in the institution in 2015 and 2016, respectively.

Anti-platelet agents are similarly known to improve the endothelial function [50]. In this study, subjects in the PED group took anti-platelet medications more frequently than those without PED at the baseline. However, the cross-sectional nature of the comparison of the baseline characteristics according to the PED status cannot show causality, and older age might result in a greater rate of prescription in the PED group.

The main limitation of this study was a small sample size, which weakened the statistical significance in the analysis of each composite secondary endpoint. In addition, considering that the proportion of CKD progression in the secondary outcome was relatively large, the significant causal effect of PED on the secondary outcome should be interpreted with caution. Despite insufficient statistical significance, the trends of each composite secondary endpoint according to the baseline RHI were consistent. Future ASCVD risk associated with PED should be validated with larger studies. In addition, this result cannot be applied in diabetic subjects without albuminuria. Lastly, CAC has been recommended for ASCVD risk-stratification [31]. As the CAC was not measured, its clinical usefulness alongside that of RHI could not be evaluated.

## Conclusions

RHI can independently predict future cardiovascular events among diabetic patients with albuminuria who are under treatment for conventional risk factors. Further studies encompassing a larger and diverse population are required for confirmation.

## Supplementary information

**Additional file 1: Table S1.** Determinants for peripheral arterial endothelial dysfunction (PED). **Table S2.** Cox Proportional Hazards Analysis for Cardiovascular Events according to Peripheral Endothelial Dysfunction (PED).

## Data Availability

Data sharing not applicable to this article as no datasets were generated or analyzed during the current study.

## Abbreviations

ACR: Albumin-to-creatinine ratio
ACEI: Angiotensin-converting enzyme inhibitor
ARB: Angiotensin receptor blocker
ASCVD: Atherosclerotic cardiovascular disease
CAC: Coronary artery calcification
CAD: Coronary artery disease
CKD: Chronic kidney disease
DBP: Diastolic blood pressure
eGFR: Estimated glomerular filtration rate
GLP-1RA: Glucagon-like peptide 1 receptor analogue
LDL: Low-density lipoprotein
MACE: Major adverse cardiovascular events
PCE: Pooled Cohort Equations
PED: Peripheral endothelial dysfunction
RH-PAT: Reactive hyperemia-peripheral arterial tonometry
RHI: Reactive hyperemia-peripheral arterial tonometry index
SBP: Systolic blood pressure
SGLT2: sodium-glucose cotransporter 2
