# Supplementary material for: Peripheral arterial endothelial dysfunction predicts future cardiovascular events in diabetic patients with albuminuria: a prospective cohort study
Source: Cardiovasc Diabetol. 2020 Jun 13;19:82. doi: 10.1186/s12933-020-01062-z (PMC7293773; doi:10.1186/s12933-020-01062-z)
Supplement: Supplementary file 1 — Additional file 1: Table S1. Determinants for peripheral arterial endothelial dysfunction (PED). Table S2. Cox Proportional Hazards Analysis for Cardiovascular Events according to Peripheral Endothelial Dysfunction (PED). [file 12933_2020_1062_MOESM1_ESM.docx]

**Additional file 1: Table S1. Determinants for peripheral arterial endothelial dysfunction (PED)**

| Variables | Odds ratio (95% CI) | *P* |
| --- | --- | --- |
| Included in the final model | | |
| Age | 1.06 (1.02, 1.11) | 0.008 |
| Systolic blood pressure | 0.96 (0.94, 0.99) | 0.006 |
| Hypertriglyceridemia | 2.20 (0.93, 5.16) | 0.074 |
| Current smoker | 2.97 (1.15, 7.63) | 0.024 |
| Not included in the final model | | |
| Sex (female) | – | 0.881 |
| BMI | – | 0.398 |
| HbA1c | – | 0.697 |
| Hypertension | – | 0.488 |
| Diastolic blood pressure | – | 0.274 |
| Anti-platelet agent | – | 0.167 |
| Cilostazol | – | 0.849 |
| Statin | – | 0.608 |

PED was defined as RHI <1.67

Backward logistic regression analysis for prediction of PED (RHI <1.67) using age, sex, BMI, HbA1c, systolic blood pressure, diastolic blood pressure, hypertriglyceridemia (triglyceridemia > 150 mg/dL), smoking history, hypertension, anti-platelet agent, cilostazol, and statin

RHI, reactive hyperemic index; BMI, body mass index

**Additional file 1: Table S2. Cox Proportional Hazards Analysis for Cardiovascular Events according to Peripheral Endothelial Dysfunction (PED)**

|  | Primary outcome | | Secondary outcome | |
| --- | --- | --- | --- | --- |
|  | HR (95% CI) | *P* | HR (95% CI) | *P* |
| Crude model |  |  |  |  |
| ASCVD-10yr risk^1^ | 11.37 (0.48, 268.83) | 0.132 | 8.50 (1.25, 57.76) | 0.029 |
| PED^2^ | 4.62 (0.60, 35.79) | 0.143 | 3.45 (1.22, 9.75) | 0.019 |
| Multivariable model^3^ |  |  |  |  |
| ASCVD-10yr risk^1^ | 8.04 (0.34, 191.95) | 0.198 | 6.55 (0.97, 44.29) | 0.054 |
| PED^3^ | 4.19 (0.54, 32.75) | 0.172 | 3.24 (1.14, 9.17) | 0.027 |

^1^Sex-specific Pooled Cohort Equation for non-Hispanic whites to estimate 10-year risk of ASCVD from the American College of Cardiology/American Heart Association [17]

^2^RHI < 1.67

^3^Cox proportional hazard analysis including ASCVD-10yr risk and RHI <1.67
